# Psychometric properties of the Family Allocentrism Scale among Japanese adults

**DOI:** 10.1016/j.heliyon.2020.e05871

**Published:** 2020-12-30

**Authors:** Yuta Ujiie, Kohske Takahashi

**Affiliations:** a Graduate School of Psychology, Chukyo University, 101-2 Yagoto Honmachi, Showa-ku, Nagoya-shi, Aichi, 466-8666, Japan; b Japan Society for the Promotion of Science, Tokyo, Japan; c Research and Development Initiative, Chuo University, 1-13-27, Kasuga, Bunkyo-ku, Tokyo, 112-8551, Japan; d Department of Psychology, Chukyo University, 101-2 Yagoto Honmachi, Showa-ku, Nagoya-shi, Aichi, 466-8666, Japan

**Keywords:** Family allocentrism–idiocentrism, Family allocentrism scale, Horizontal and vertical individualism-collectivism

## Abstract

The Family Allocentrism Scale (FAS) was developed to assess individual differences in allocentrism–idiocentrism with reference to the family. To date, no prior study has adequately investigated the psychometric properties of the Japanese version of the FAS in Japanese samples, although Japanese culture is considered as a symbol of an interdependent (or collectivist) culture. This study attempted to demonstrate the validity of the factor structure and the convergent validity of the Japanese version of the FAS in a sample of Japanese adults. The results of the confirmatory factor analysis showed a lack of fit of the one-factor model for all FAS items but the fit improved to the acceptable level if some items with low factor loadings were removed. The internal consistency measure (Cronbach's alpha) of the FAS indicated an acceptable level of reliability. The results also showed that the FAS scores were closely related to the scores of horizontal collectivism, vertical collectivism, and interdependence. Our findings indicate the validity and reliability of the Japanese version of the FAS, thereby providing a validated tool for the investigation of cross-cultural differences in family allocentrism–idiocentrism.

## 1 Introduction

### 1.1 Collectivism and individualism in cultures

The diversity of behavioral and cognitive patterns in different cultures has been discussed within the framework of the opposing cultural patterns of collectivism and individualism (e.g., Singelis et al. 1995; Triandis and Gelfand, 1998). It has been shown that people in collectivist cultures are interdependent within their in-groups (e.g., family), give priority to the goals of their in-group, and are concerned with maintaining their relationship with their in-group, whereas people in individualist cultures are independent from their in-group and give priority to their personal goals (e.g., Lay et al., 1998; Sato, 2007; Triandis, 1989, 2001). A similar framework is interdependence/independence, which is also widely used to explain the divergence in self-construal between cultures (e.g., Markus and Kitayama, 1991; Ohashi, 2004). Independence involves the conception of the self as autonomous and self-determined, and this conception is relevant to the view of the self in American culture as well as many Western cultures. Interdependence implies that the conception of the self is not separate from the social context but is more connected and less differentiated from others (Markus and Kitayama, 1991; Na et al., 2010; Nisbett and Masuda, 2003). Such a tendency is common among Japanese (Markus and Kitayama, 1991).

### 1.2 Family Allocentrism Scale

The Family Allocentrism Scale (FAS) is a self-report questionnaire developed by Lay et al. (1998) to assess individual differences in idiocentrism and allocentrism. The terms idiocentrism and allocentrism are defined as the degree of collectivism and individualism at an individual level, respectively (Triandis, 1989). The FAS focuses on one's emotional and cognitive relatedness with one's family because family is considered the most salient in-group in cross-cultural studies of self-construal (Lay et al., 1998; Markus and Kitayama, 1991). This scale, which assumes a one-factor structure, consists of six idiocentric-worded items and 15 allocentric-worded items, which were selected from the item analyses of data collected from samples in Eastern and Western cultures (Lay et al., 1998). This scale has been translated into several languages and is widely used in cross-cultural studies (e.g., in Chinese by Li et al., 2018; in German by Keller et al., 2006; in Italian by Li et al., 2018; and in Portuguese by Seidl-de-Moura et al., 2013).

### 1.3 The convergent validity of the FAS

The convergent validity of the FAS was demonstrated by Sato (2007) to examine relationships with two other scales associated with collectivism/individualism, the horizontal and vertical individualism-collectivism scale (HVIC, Singelis et al., 1995) and Self-Construal Scale (SCS, Singelis, 1994). The HVIC measures individual differences in horizontal and vertical individualism/collectivism (Singelis et al., 1995). The scale was developed based on the concept that collectivism/individualism may be further defined according to four attributes by adding a dimension to indicate whether a culture emphasizes equality (horizontal) or hierarchy (vertical) (e.g., Komarraju and Cokley, 2008; Triandis, 2001). For instance, horizontal collectivism emphasizes the self as a part of a group, while vertical collectivism emphasizes group achievement and competition with other groups (Sato, 2007). The SCS measures individual differences in both independent and interdependent self-construals (Markus and Kitayama, 1991). Sato (2007) administered the FAS, HVIC, and SCS to Canadian adults and examined the relationships among the scales. The results showed that high FAS scores were associated with high levels of horizontal and vertical collectivism as well as high interdependence in one's self-construal. These findings indicate the convergent validity of FAS, and also demonstrate that the FAS scores are more closely related to the collectivistic than the individualistic aspects of collectivism/individualism.

### 1.4 The validity of the factor structure

While the available evidence supports the convergent validity of the FAS, little evidence supports the validity of its factor structure. To date, the FAS has been assumed to fit a one-factor model (e.g., Keller et al., 2006; Lay et al., 1998), although some studies failed to demonstrate the goodness of model fit for a one-factor structure (Li et al., 2018; Seidl-de-Moura et al., 2013). Seidl-de-Moura et al. (2013) tested the factor structure of the FAS using its Portuguese version in a Brazilian sample. The results of principal factor analysis showed that the two-factor model was the best solution, rather than the one-factor model found in the original study (Lay et al., 1998). In their results, Seidl-de-Moura et al. (2013) found that the allocentric-worded items loaded on factor 1, while the idiocentric-worded items loaded on factor 2. Li et al. (2018) tested the factor structure of the FAS among Chinese and Italian samples using the FAS Chinese and Italian versions, respectively. The results of the confirmatory factor analysis showed that not all items were significantly loaded on the latent factor; in particular, the factor loadings of some items, which were mainly idiocentric-worded items (Lay et al., 1998), were low or insignificant. Li et al. (2018) noted the problem that some of the FAS items are ambiguously worded (i.e., the idiocentric items) and may result in low factor loadings (Li et al., 2018). These results suggest that if the FAS is assumed to have a one-factor structure, the scores reflect levels of family allocentrism unless the ambiguously worded idiocentric items are rephrased. Indeed, Sato (2007) showed that the FAS reflected collectivistic (i.e., allocentric) aspects rather than individualistic (i.e., idiocentric) aspects of collectivism/individualism.

### 1.5 The purpose of this study

To date, no prior study has adequately investigated the psychometric properties of the Japanese version of the FAS in Japanese samples, although Japanese culture is considered symbolic of an interdependent (or collectivist) culture (e.g., Markus and Kitayama, 1991). Hence, this study attempted to demonstrate evidence for the validity of the factor structure and the convergent validity of the Japanese version of the FAS in a sample of Japanese adults. In our study, we first tested the factor structure of the FAS by using confirmatory factor analysis, as in Li et al. (2018). We also evaluated the reliability and score distribution of the FAS. Second, we tested the convergent validity of the FAS by examining its relationship with other measures associated with collectivism/individualism. With reference to Sato (2007), we used the HVIC scale and Independent and Interdependent Self-Construal Scale. We hypothesized that FAS scores are closely related to the scores of horizontal collectivism, vertical collectivism, and interdependence, as reported by Sato (2007).

## 2 Methods^1^

### 2.1 Participants

The sample consisted of 600 adults (300 men and 300 women), all of whom were native Japanese speakers. The mean age was 34.5 years (SD = 8.61). Our sample size was determined by referring to previous studies that had similar purposes as the current study, which was to examine the validity of the factor structure of questionnaire data (e.g., Henson and Roberts, 2006; MacCallum et al., 1999). All participants were recruited online and participated in the study voluntarily. Ethical approval for this study was obtained from the Ethical Committee of Chukyo University. Participants provided written informed consent for their participation and for publication in an online open-access platform.

### 2.2 Instruments

#### 2.2.1 The Japanese version of the FAS (Lay et al., 1998)

We used the Japanese version of the FAS (Lay et al., 1998) after translating the scale from English to Japanese. First, an English–Japanese bilingual speaker translated the original items from the English version of the FAS to Japanese. Then, another English–Japanese bilingual speaker, who was blinded to the original version of the items, translated the Japanese items into English. Finally, a third bilingual speaker checked the Japanese items as well as the back-translated items to ensure they corresponded with the original meanings of the FAS items. The final version of the Japanese FAS contains 21 items on a 5-point Likert-scale ranging from “strongly disagree” to “strongly agree.” This scale is similar to the original English version (Lay et al., 1998).

#### 2.2.2 The Japanese version of the horizontal and vertical individualism-collectivism scale (Ohashi, 2004; Singelis et al., 1995)

We used the Japanese version of the HVIC (Ohashi, 2004). This scale contains four independent measures (i.e., horizontal individualism, horizontal collectivism, vertical individualism, and vertical collectivism), each of which consists of eight items on a 9-point Likert scale ranging from “definitely no” to “definitely yes.”

#### 2.2.3 Independent and interdependent Self-Construal Scale (Takata, 1999)

We also used the Independent and Interdependent Self-Construal Scale (IISC; Takata, 1999). This scale assesses the degree of independence (or interdependence) in an individual's self-construal (Takata, 1999) using 10 independent items and 10 interdependent items rated on a 7-point Likert scale ranging from “definitely no” to “definitely yes.”

### 2.3 Procedure

In this study, we obtained questionnaire data from Japanese adults online. All participants completed all questionnaires: Japanese versions of the FAS (Lay et al., 1998), HVIC (Ohashi, 2004; Singelis et al., 1995), and the IISC (Takata, 1999). In total, data from 600 adults were analyzed.

### 2.4 Analysis

Statistical analysis was conducted using R Version 1.2.1335 for Windows (R Foundation for Statistical Computing, Vienna, Austria). We performed confirmatory factor analyses (CFA) to confirm the validity of the one-factor structure of the FAS (Li et al., 2018) using the R lavaan package (Rosseel, 2012). To judge the model fit, we used the following fit indexes: the standardized root-mean-square residual (SRMR), the root-mean-square error of approximation (RMSEA), and the comparative fit index (CFI) (e.g., Hu and Bentler, 1999). We set the cutoff value of each fit index to judge the goodness of model fit (one-factor model) as follows: a cutoff value less than .08 for SRMR, a cutoff value less than .06 for RMSEA, and a cutoff value more than .90 for CFI (e.g., Cheung and Rensvold, 2002; Hu and Bentler, 1999). We also evaluated the distribution of the FAS scores using the Kolmogorov-Smirnov test, and the reliability of the FAS scores by calculating Cronbach's alpha. In addition, we analyzed the relationships among the FAS, HVIC, and IISC by calculating correlation coefficients.

## 3 Results

### 3.1 Factor structure and descriptive statistics of the Japanese version of the FAS

#### 3.1.1 Factor structure of the FAS

First, we performed CFA to confirm the validity of the one-factor structure of the FAS (Li et al., 2018). Table 1 shows the model fit indexes of the one-factor structure of the scale. The results showed that the goodness of model fit for the original one-factor model of the FAS (21 items) did not reach acceptable levels; the CFI was lower than .90, the RMSEA was greater than .06, and the SRMR was greater than .08.

**Table 1:** Results of confirmatory factor analysis of the FAS (one-factor model).

Models	χ^2^	*df*	CFI	RMSEA	SRMR	AIC
one-factor model (21 items)	1235.651	189	.715	.096	.085	32736.39
one-factor model (15 items)	496.244	90	.862	.087	.058	22919.54

Note: *χ*^*2*^ = the statistical value of chi-square test, *df* = degree of freedom, CFI = comparative fit index, RMSEA = root-mean-square error of approximation, SRMR = standardized root-mean-square residual, and AIC = Akaike Information Criterion.

Then, we calculated the standardized factor loadings to confirm the magnitude of the factor loadings. Table 2 shows the standardized factor loadings for each item. The results revealed that six items (i.e., items 3, 6, 15, 18, 19, and 21) showed factor loadings that were lower than 0.40. Moreover, these same six items also showed lower loadings in the one-factor model of the FAS among Chinese and Italian samples (Li et al., 2018). Therefore, we removed these six items and carried out the confirmatory factor analysis with the maximum likelihood method for the one-factor model by using the scores from the remaining 15 items. As a result, the goodness of model fit indices was improved compared to the original one-factor model (in Table 1); the CFI was close to .90, which indicates a moderate level of goodness of model fit, the SRMR was less than .08, and the RMSEA also decreased, although it was greater than the cutoff score of .06. In addition, we conducted exploratory factor analysis (EFA) to confirm whether the six excluded items converge on another latent factor^2^. The results showed that the 15 items remaining by CFA were loaded onto Factor 1, while four of the six excluded items were loaded onto Factor 2. According to the results, Factors 1 and 2 could be interpreted as allocentric and idiocentric dimensions, respectively. Such a two-factor model failed to converge all items of the FAS, as shown in Table 3; that is, the second latent factor (Factor 2) did not completely converge all the six excluded items. Considering these results, in the following analysis, we adopted the total score of all the 15 items as the total FAS score, in accordance with the assumption of the one-factor model in the original study of the FAS (Lay et al., 1998).

**Table 2:** Mean, SD, and standardized factor loading of each item.

No.	Item	Mean	SD	Standardized factor loadings
1	I am very similar to my parents.	3.2	.99	**.41**
2	I work hard at school to please my family.	2.7	1.09	**.41**
3	I follow my feelings even if it makes my parents unhappy. (R)	3.1	.96	.21
4	I would be honored by my family's accomplishments.	3.3	1.01	**.58**
5	My ability to relate to my family is a sign of my competence as a mature person.	3.5	.95	**.52**
6	Once you get married your parents should no longer be involved in major life choices. (R)	3.1	1.00	.28
7	The opinions of my family are important to me.	3.5	.94	**.69**
8	Knowing that I need to rely on my family makes me happy.	2.9	.95	**.57**
9	I will be responsible for taking care of my aging parents.	3.5	.99	**.66**
10	If a family member fails, I feel responsible.	3.0	.98	**.55**
11	Even when away from home, I should consider my parents' values.	3.8	1.06	**.71**
12	I would feel ashamed if I told my parents “no” when they asked me to do something.	3.0	1.00	**.59**
13	My happiness depends on the happiness of my family.	3.3	1.00	**.72**
14	I have certain duties and obligations in my family.	3.1	1.04	**.58**
15	There are a lot of differences between me and other members of my family. (R)	2.8	.94	.01
16	I think it is important to get along with my family at all costs.	3.3	.99	**.64**
17	I should not say what is on my mind in case it upsets my family.	3.1	.99	**.47**
18	My needs are not the same as my family's. (R)	2.4	.93	-.07
19	After I leave my parents' house, I am not accountable to them. (R)	3.3	1.03	.20
20	I respect my parents' wishes even if they are not my own.	2.9	.93	**.57**
21	It is important to feel independent of one's family. (R)	2.6	.89	-.14

Note: Values of items that standardized factor loadings are greater than .40 and are in bold. (R) indicates reversely scored items, which were reversely scored before performing the CFA.

**Table 3:** Oblimin-rotated two-factor solution for all items of the FAS.

Items	Factor 1	Factor 2	*h*^*2*^	*u*^*2*^
1	**.41**	-.06	.17	.83
2	**.40**	.11	.18	.82
3	.21	**.46**	.26	.74
4	**.59**	-.03	.34	.66
5	**.53**	-.24	.34	.66
6	.29	.35	.20	.80
7	**.70**	.01	.48	.52
8	**.56**	.14	.34	.66
9	**.66**	-.08	.44	.56
10	**.54**	.01	.30	.70
11	**.72**	-.17	.55	.45
12	**.58**	.02	.34	.66
13	**.71**	.13	.53	.47
14	**.58**	-.07	.34	.66
15	.01	**.56**	.31	.69
16	**.64**	.12	.43	.57
17	**.47**	-.10	.23	.77
18	-.08	**.55**	.31	.69
19	.20	.28	.12	.88
20	**.57**	.12	.34	.66
21	-.16	**.50**	.27	.73
Variance	.25	.07		

Note: Items with loadings above .40 are in bold.

#### 3.1.2 The reliability and descriptive statistics of FAS

A summary of the 15 items of the FAS is shown in Table 4. The kurtosis and skewness of the FAS scores were 1.52 and -.54, respectively. We evaluated the distribution of the FAS scores using the Kolmogorov-Smirnov test and the reliability of the FAS by calculating Cronbach's alpha. The Kolmogorov-Smirnov test showed that the FAS scores were not normally distributed (p < .05). However, the internal consistency measure (Cronbach's alpha) for the FAS (*α* = .88) indicated an acceptable level of reliability. The results for the other two scales (i.e., the HVIC and IISC) are also summarized in Table 3.

**Table 4:** Summary of results for the FAS, HVIC, and IISC.

Scales	items	Mean	SD	Kurtosis	Skewness	Cronbach's α
1. FAS	15	48.0	9.18	1.52	-.54	.88
2. HVIC						
HI	8	44.6	9.12	.69	.34	.83
VI	8	37.6	7.65	1.79	-.05	.70
HC	8	41.7	10.07	1.34	-.15	.87
VC	8	38.3	8.38	1.84	-.45	.77
3. IISC						
Interdependence	10	45.2	7.89	1.27	.16	.83
Independence	10	43.8	8.41	.72	-.23	.86

Note: FAS = Family Allocentrism Scale, HVIC = Horizontal and Vertical Individualism-Collectivism Scale, HI = Horizontal Individualism, VI = Vertical Individualism, HC = Horizontal Collectivism, VC = Vertical Collectivism, and IISC = Independent and Interdependent Self-Construal Scale. Interdependence and independence are subscales of the IISC.

### 3.2 Correlations between the FAS, HVIC, and IISC

To examine the relationships between the FAS, HVIC, and IISC, we calculated Spearman's correlation coefficients between the FAS score and the subscales of the HVIC and IISC (shown in Table 5). The correlation analysis showed that the FAS score was positively and significantly correlated with the scores of horizontal collectivism (HC), vertical collectivism (VC), and interdependence (the IISC subscale). These results indicate that the FAS score was associated with all variables that assess the levels of individuals' collectivism. The analysis also showed a positive correlation between the FAS score and horizontal individualism and vertical individualism, although the magnitudes of these effect sizes were small (|*r* | < .02) (e.g., Algina and Keselman, 1999; Cohen, 1988).

**Table 5:** Correlations between total FAS score and the subscale scores of the HVIC and IISC.

Scales	2	3	4	5	6	7
1. FAS	.15 ∗∗	.17 ∗∗	.41 ∗∗	.50 ∗∗	.23 ∗∗	.01
2. HI		.29 ∗∗	.31 ∗∗	.17 ∗∗	-.03	.39 ∗∗
3. VI			.13 ∗∗	.28 ∗∗	-.03	-.03
4. HC				.45 ∗∗	.16 ∗∗	.06
5. VC					.15 ∗∗	-.18 ∗∗
6. Interdependence						.06
7. Independence						-

Note: ∗∗*p* < .01.

In addition, we found that the interdependence score correlated positively with the scores of HC and VC. We also found that the independence score correlated positively with the horizontal individualism (HI) score. These results indicate that self-construal of interdependence is similar to individual traits associated with collectivism, while self-construal of independence is similar to individual traits associated with individualism.

## 4 Discussion

In this study, we attempted to confirm the validity of the one-factor structure assumed in the original study of the FAS (Lay et al., 1998) by assuming that all items loaded on one factor. The results of the CFA showed a lack of fit of the one-factor model for all FAS items, however, the fit improved to the acceptable level if some items with low factor loadings were removed. The internal consistency measure (Cronbach's alpha) of the FAS indicated an acceptable level of reliability. In addition, we tested the convergent validity of the FAS by examining relationships with the HVIC and IISC, as reported in a previous study (Sato, 2007). Our results replicated the results of Sato (2007) showing that as we expected, the FAS scores are closely related to the scores of HC, VC, and interdependence. To our knowledge, our study is the first investigation to adequately examine the psychometric properties of the Japanese version of the FAS.

Our results indicate that all FAS items did not converge for the one-factor model, consistent with the results of previous studies (Li et al., 2018; Seidl-de-Moura et al., 2013). This lack of convergence may be due to the wording and structure of the FAS, 15 allocentric-worded items and six idiocentric-worded items (Lay et al., 1998). Indeed, our CFA results showed that the factor loadings on six items, which are all idiocentric-worded items, were lower than .40. Previous studies have also showed that, in common across different samples, all allocentric-worded items were converged on one latent factor, however, idiocentric-worded items were not (Li et al., 2018; Seidl-de-Moura et al., 2013). These results suggest that if FAS has a one-factor structure, it may be appropriate to consider the total FAS score as reflecting collectivistic (i.e., allocentric) aspects at a personal level, rather than individualistic (i.e., idiocentric) aspects.

Our results also demonstrated the convergent validity of the Japanese version of the FAS, as shown in Sato (2007). The FAS scores were significantly and positively correlated with the scores for HC, VC, and interdependence of self-construal. This finding indicates that people with high levels of family-allocentrism also have interdependence within their in-groups, give priority to the goals of the in-group, and are concerned with maintaining their relationship with the in-group (e.g., Lay et al., 1998; Sato, 2007; Triandis, 1989; 2001). Our results provide evidence for the construct validity of the FAS, with respect to individual differences in collectivism at the person level (Sato, 2007). However, our results failed to find evidence with respect to individual differences in individualism. In a previous study (Sato, 2007), the total FAS score was negatively correlated with HI and independent self-construal. A possible reason for the null results in this study may be due to the heterogeneity of our Japanese sample. That is, the scores for the individualism scales were not normally distributed, which may be because there are relatively few people with high individualism in Japanese culture (Markus and Kitayama, 1991). In a previous study, the sample consisted of people from different cultures and ethnicities, and included both collectivists and individualists (Sato, 2007). Even with such a diverse sample (Sato, 2007), the effect sizes of the correlations were small (|r | <.20) (Cohen, 1988). These findings suggest that the relationship between the FAS and traits associated with individualism is present, but it is not robust enough to find it in a heterogeneous sample from a collectivism culture.

A cross-cultural comparison of FAS with our data also confirms the validity of the definition of collectivism/individualism used in previous cross-cultural studies (e.g., Markus and Kitayama, 1991; Nisbett and Masuda, 2003). Most cross-cultural studies have applied the dichotomous structure between Western and East Asian cultures; that is, American culture is symbolic of the individualism culture, and Japanese culture is symbolic of the collectivist culture (Markus and Kitayama, 1991). Such a definition is arbitrary and often lacks confirmatory evidence. The comparison of FAS scores can address this issue; that is, cross-sectional research using the FAS in multiple cultures can confirm whether each culture lies on a continuum distribution of collectivism. Indeed, the previous study confirmed the operational definition of collectivism/individualism, i.e., whether, the culture is defined as an independent culture or an interdependent culture, by using the FAS (Keller et al., 2006).

Meanwhile, our results also raise a concern whether the scores from all the original items should be interpreted as levels of family allocentrism in future studies. Once again, our results of CFA should be highlighted as 15-items of the original items loaded on one latent factor indicating the axis of levels of family allocentrism, similar to previous studies (Li et al., 2018; Seidl-de-Moura et al., 2013). These results suggest the need for a further scrutiny of FAS in terms of wording and item structure. Moreover, a future study should determine whether scores of the 15 items or original 21 items could be used as the total FAS score, after conducting CFA.

There are some limitations to this study. First, the assumption of the factor structure of the FAS could not be validated completely. Although our study assumed the one-factor model based on the original study, another possibility also remains that the factor structure of the FAS may be bi-dimensional. This means that the FAS contains two factors of allocentric and idiocentric dimensions. Indeed, our EFA results exhibited limited support for the two-factor model since 15 allocentric-worded items converged on the allocentric factor (Factor 1); however, some of the six idiocentric-worded items did not converge on the idiocentric factor (Factor 2). If such insufficient results are indeed due to the wordings of the above items, then the fit of the two-factor model may be improved by rephrasing the idiocentric-worded items.

Second, the absence of correlation between the total FAS score and Independence scores may depend on the difference in the scales used in the present and the previous studies. Sato (2007) used the Self-Construal Scale, developed by Singelis (1994), to assess the degree of Independence and found significantly negative correlation with the FAS scores (*r* = -.13). In this study, we used the Independent and Interdependent Self-Construal Scale (Takata, 1999), which is different from Singelis' scale yet normalized for the Japanese sample (Takata, 1999). Although both scales were developed to assess the degree of Independence in an individual's self-construal (Singelis, 1994; Takata, 1999), the wordings and structure (e.g., the number of items) were different to some extent. Therefore, such differences may have caused inconsistency between the current and the previous study's results (Sato, 2007) in terms of the correlation between the FAS and Independence scores. Future studies should investigate these issues.

## 5 Conclusion

In summary, the current study demonstrated evidence for the validity of the factor structure and the convergent validity of the Japanese version of the FAS in a sample of Japanese adults. Our results indicate that the Japanese version of the FAS can measure individual differences in allocentrism as well as the other language versions of FAS (e.g., Lay et al., 1998; Li et al., 2018; Seidl-de-Moura et al., 2013). Given our findings, the next step is to test the total FAS score among multiple cultures and confirm whether and how each culture lies on a continuum distribution of collectivism.

## Declarations

### Author contribution statement

Y. Ujiie: Conceived and designed the experiments; Performed the experiments; Analyzed and interpreted the data; Contributed reagents, materials, analysis tools or data; Wrote the paper.

K. Takahashi: Conceived and designed the experiments; Analyzed and interpreted the data; Wrote the paper.

### Funding statement

This work was supported by a Grant-in-Aid for Early-Career Scientists (Grant No. 19K20650), the Promotion of Joint International Research (Grant No. 20KK0054), and a Grant-in-Aid for JSPS Fellows (Grant No. 19J00722). This work was also supported by a Grant-in-Aid for Scientific Research on Innovative Areas “Construction of the Face- Body Studies in Transcultural Conditions” (Grant No. 17H06342).

### Data availability statement

Data associated with this study has been deposited at the Centre for Open Science: https://osf.io/k953m/.

### Declaration of interests statement

The authors declare no conflict of interest.

### Additional information

No additional information is available for this paper.

## Footnotes

1 The methods were preregistered on Open Science Framework (https://osf.io/djt97).

2 This analysis is not pre-registered.
